# Spectrum of Renal Lesions on Autopsy: Experience of a Tertiary Level Institute Based on Retrospective Histopathological Analysis

**DOI:** 10.7759/cureus.17064

**Published:** 2021-08-10

**Authors:** Pratima Khare, Renu Gupta, Swapnil Agarwal, Avni Bhatnagar, Rajani Anand

**Affiliations:** 1 Pathology, Dr Baba Saheb Ambedkar (BSA) Medical College and Hospital, New Delhi, IND

**Keywords:** autopsy, renal histopathology, tubules, interstitial, glomeruli

## Abstract

Introduction: Last few decades have seen a remarkable increase in the elderly population. Aging is an established risk factor for chronic kidney diseases associated with increased mortality and morbidity. The frequency and spectrum of renal pathology on autopsy specimen is not well documented and is often overlooked by physicians as well as forensic pathologists.

Aim: The present study aims to find out the prevalence and pattern of various types of renal pathologies, based on the histopathological analysis of renal tissue where autopsies were performed whether related or unrelated to renal diseases.

Material and methods: This retrospective study of six years consisted of 557 autopsies. The bits of 417 samples of kidney tissue/whole kidney, retrieved at the time of autopsy were received, processed, and examined.

Results: The male to female ratio approximately was 2:1, and 83.69% of cases were in 11-50 years age groups. Among the 159 cases having definitive renal findings, the commonest pathologies were seen in tubules and interstitial tissues (58.49% cases), followed by 38.36% cases with involvement of all components of renal tissues. There were four cases of isolated vascular changes and one case having Hodgkin’s lymphoma. The series had 12 cases of renal tuberculosis.

Conclusions: The study highlights the various lesions of kidney found in renal tissue obtained on autopsy. The physician as well as forensic pathologists must be aware of the high prevalence and wide spectrum of possible pathologies in the kidney. The focus should be to develop more efficacious diagnostic methods for timely intervention.

## Introduction

Autopsy is an essential legal procedure in all medicolegal deaths and is regarded as the gold standard for determining the cause of death and confirming or refuting pre-mortem diagnosis. It is also an important tool for retrospective quality assessment of the clinical diagnosis and serves as an educational tool to the clinician [1]. In hospital deaths, it also provides additional information about complications of intensive care and information about existing co-morbidities that could have affected the recovery of the patient. Despite pitfalls like delay in carrying out autopsy, improper sampling, improper preservation, transport, and non-availability of representative samples, the autopsy is still very useful for studying the disease process in situ, thus enriching knowledge.

Incidence of biopsy/autopsy proven cases of renal pathology in population varies in different parts of the world [2]. The kidneys are examined for diseases, injury, and other related changes suggesting the cause of death or the incidental findings in many situations. Since kidney biopsy is usually avoided in critically ill patients, histologic evaluation of kidneys may be the first and only opportunity to identify these diseases [3]. The development of national/regional registers may help to improve knowledge about renal pathologies prevalent in that particular region and to devise effective measures towards proper scientific management of these illnesses [2].

## Materials and methods

The present retrospective study, conducted in the Department of Pathology, included all the consecutive post-mortem cases from January 2015 to December 2020. In view of the medicolegal nature and legal bonding as per law, no ethical clearance for conducting the autopsy and carrying out the study was required. During the said period, a total of 557 autopsies were conducted that included 306 (54.93%) cases brought dead under different circumstances like accidents, suicide, homicide, foeticide, hanging, drowning, burns, electrocution, abandoned dead bodies, snake bite, and cases with suspicion of foul play in the cause of death. The remaining 251 (43.07%) cases were hospital deaths which included cases admitted with medicolegal problems or with alleged medicolegal negligence. The autopsy time varied from one day to several days depending upon receiving a dead body in the autopsy surgical theatre.

All 54 cases of autopsies where kidney tissue was not received for histopathology examination and 86 cases of autolyzed kidney tissue were excluded in this study.

After exclusion criteria, the histopathology slides of the kidney tissues of the remaining 417 autopsy cases were reviewed. The staining agent used in most cases was H&E. Periodic acid-Schiff (PAS) stain was used wherever required. The histopathological slides were first reviewed by a team of three pathologists. Subsequently, the final review was done by a team of two different pathologists with expertise in autopsy histopathology. The clinical data included patient’s age, gender, history wherever available, clinical presentation in cases of the institutional deaths, contributing cause of death, probable time interval between death and post-mortem, and state of important organs like lung and liver, besides kidney at the time of post-mortem.

## Results

The series had 268 (64.26%) males and 149 (35.74%) females (Table 1). The age varied from newborn to 86 years, with the maximum number of cases (118 males and 79 females) falling within the age group of 11-30 years. The age group of 31-50 years had the second highest number of cases (111 males and 41 females). Combined together, these two groups accounted for 83.69% of cases.

**Table 1:** Renal lesions age and sex distribution (n=417) M: male; F: female

Histopathology	Age group (%)										
	< 10 years n=24 (5.75)		11-30 years n=197 (47.24)		31-50 years n=152 (36.45)		51-70 years n=34 (8.15)		>70 years n=10 (2.39)		Total n=417 (100)
	M	F	M	F	M	F	M	F	M	F	
No renal pathology	2	4	23	12	15	6	3	1	1	--	67 (16.06)
Only congestion	7	7	59	33	60	13	9	1	0	2	191 (45.80)
Predominant involvement of tubular & interstitial	1	2	29	25	19	12	4	1	0	0	93 (22.30)
Involvement of all components	0	0	7	8	17	10	7	5	0	0	61 (14.62)
Vascular lesions	0	1	0	0	0	0	3	0	0	0	4 (0.95)
Neoplasm & other	0	0	0	1	0	0	0	0	0	0	1 (0.25)
Total cases (M)	10	--	118	--	111	--	26	--	3	--	268 (64.26)
Total cases (F)	--	14	--	79	--	41	--	8	--	7	149 (35.74)

The series had 67 cases (16.06%) without any remarkable pathology in the kidney tissue. Congestive changes, a non-specific common feature in autopsies in various organs, were found in the renal tissue in 191 cases (45.80%). These cases were not further analyzed. After excluding these 258 cases (67 and 191 cases), the remaining 159 cases with definitive kidney lesions were examined. The common pathologies were the predominant involvement of tubular and interstitial components in 93 (22.30%) cases. There were 61 (14.62%) cases that had involvements of all components of renal tissue. There were four (0.95%) cases with only vascular lesions in the series and one case (0.25%) of Hodgkin’s lymphoma.

**Figure 1:**
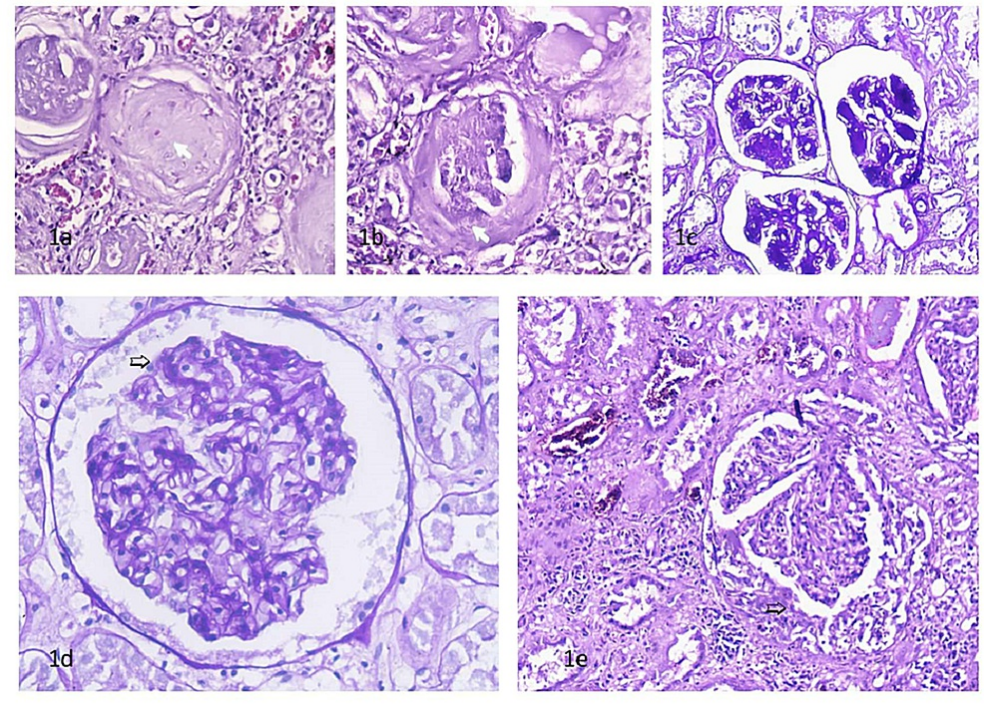
Global glomerulosclerosis H&E 10x20 (a), segmental glomerulosclerosis H&E 10x20 (b), nodular glomerulosclerosis PAS 10x20 (c), basement membrane thickening PAS 10x40 (d), and crescentic glomerulonephritis H&E 10x20 (e) PAS: periodic acid-Schiff

Among these 159 cases with definitive renal lesions, 61 (38.36%) cases had involvement of all the components of renal tissues with changes suggestive of chronic kidney disease (Table 2). Four of them showed diffuse glomerular involvement and 57 cases had focal involvement of glomeruli. The Glomeruli showed global glomerulosclerosis in 20 cases (Figure 1a), segmental glomerulosclerosis in four cases (Figure 1b), and nodular glomerulosclerosis in six cases (Figure 1c). The presence of both global (complete sclerosis of glomeruli) and segmental sclerosis (sclerosis of a part of glomeruli) was observed in 27 cases. Basement membrane thickening was present in 21 cases (Figure 1d). The commonest pathology was global and segmental combined in 27 cases, followed by global changes in 20 cases. There were two cases of glomerulonephritis with crescent formation (Figure 1e). These 61 cases also showed tubulointerstitial changes in form of RBC cast, tubular atrophy, thyroidization, interstitial fibrosis, and chronic inflammatory cell infiltrate. Vascular changes in the form of thickening of tunica media were seen.

**Table 2:** Types of renal lesions (n=159)

Major pathology	Cases (%)	Types of lesions detected
Involvement of all component	61 (38.36)	Chronic kidney disease: glomerulosclerosis (like global {20}; segmental {4}; nodular {6}; both global and segmental {27}; basement thickening {21}); glomerulonephritis with crescent formation (2); all 61 cases had tubular interstitial changes (tubular atrophy, thyroidization, interstitial fibrosis, chronic inflammatory cell infiltrate) and vascular changes.
Predominant involvement of tubules and interstitial (T&I)	93 (58.49)	Hydropic changes (50); chronic pyelonephritis (45); tubular necrosis (24); tuberculosis (12); chronic inflammation and abscess (7).
Mainly vascular change	04 (2.51)	Thickening of tunica media (4)
Neoplastic	01 (0.62)	Hodgkin’s lymphoma (1)

**Figure 2:**
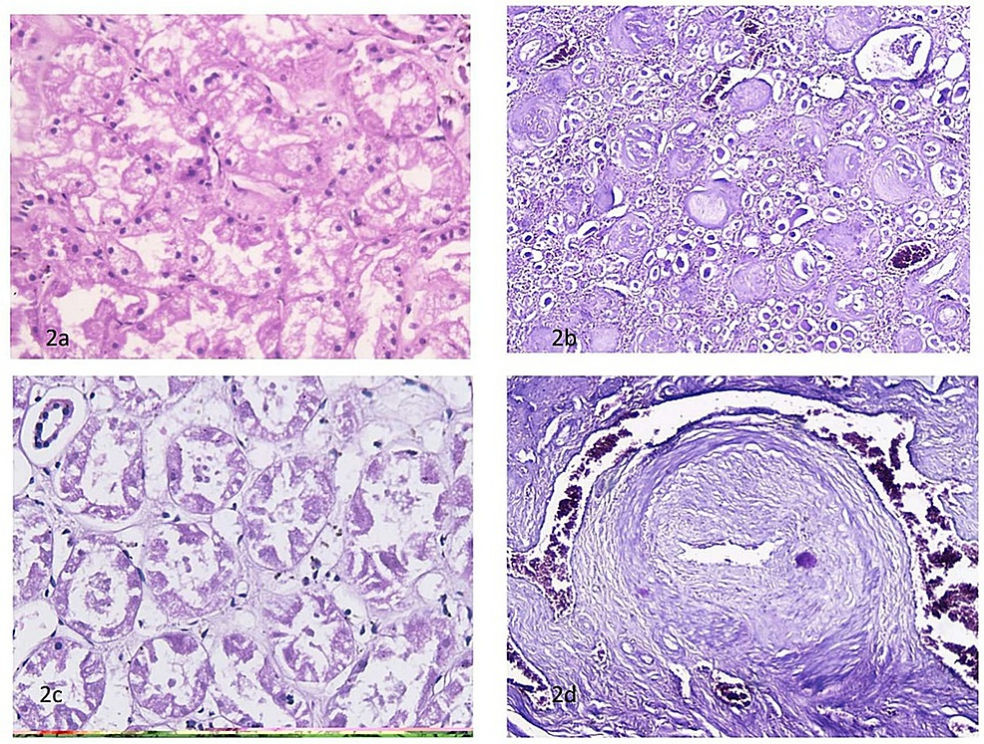
Hydropic degeneration H&E 10x20 (a), chronic pyelonephritis H&E 10x10 (b), acute tubular necrosis H&E 10x20 (c), and blood vessels showing thickening of tunica media H&E 10x20 (d)

**Figure 3:**
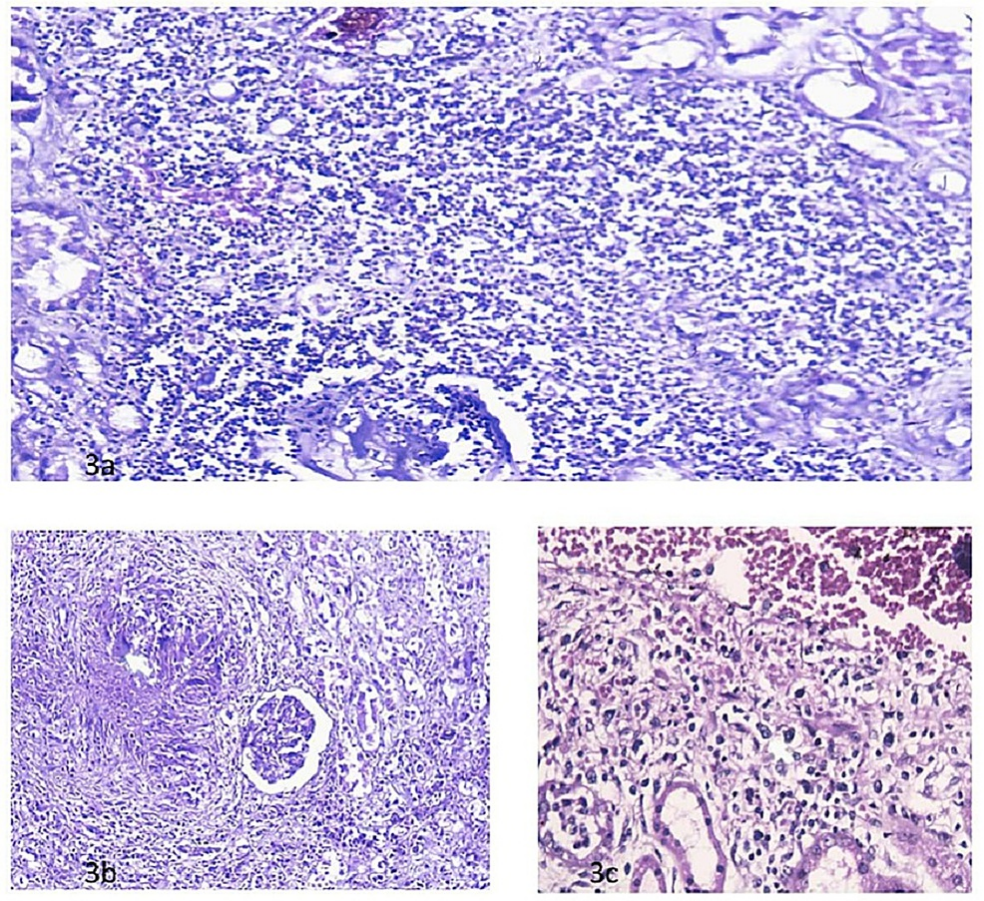
Chronic inflammatory infiltrate H&E 10x2 (a), tubercular granuloma and necrosis H&E 10x20 (b), and Hodgkin’s lymphoma (atypical mononuclear cells) with bi-nucleate cells H&E 10x40 (c)

Among the 93 (58.49%) cases with predominant features present in tubular and interstitial tissues, the commonest feature was the presence of hydropic changes in 50 cases (Figure 2a). Chronic pyelonephritis was the second commonest pathology seen in 45 cases (Figure 2b). There were 24 cases of acute tubular necrosis, 12 cases of tuberculosis, and seven cases of chronic inflammation and abscess (Figures 2c, 3a).

There were four (2.51%) cases of isolated vascular lesions with thickening of tunica media (Figure 2d). Among these, there were three males above the age of 50 years whereas there was one female under 10 years of age. There was only one case (0.62%) of Hodgkin’s lymphoma in the renal tissue present in our series (Figure 3c).

There were 12 cases of granulomatous lesions in renal tissue (Table 3). Among them, six were males and six were females. There were nine cases in the age group of 11-30 years and three in the age group of 31-50 years. All these cases had caseous necrosis and epithelioid cell granulomas (Figure 3b). The diagnosis of tuberculosis was made based on these findings. However, no acid-fast bacilli (AFB) was seen on Ziehl-Neelsen (ZN) staining in any of these cases. Our series had 57 cases with evidence of tuberculosis on post-mortem in different parts of the body including lung tuberculosis in 47 cases. However, among the 12 cases with renal tuberculosis, only two cases had co-existing lung tuberculosis while one had abdominal tuberculosis whereas in nine cases (75%) no extra-renal tubercular lesions were detectable. Among those nine cases, there was one case of simple congestive changes present in renal tissue, one case of focal hydro-nephrosis with interstitial fibrosis, and one case of glomerulosclerosis with changes in the tubule. There were two cases with additional hydropic changes in the tubule whereas four cases had no additional renal pathology except for the presence of granuloma. Among the two cases with associated lung tuberculosis, besides the presence of granulomas in renal tissue, one had evidence of focal tubular necrosis and the other had focal hydro-nephrosis with interstitial fibrosis. The case of renal tuberculosis with associated abdominal tuberculosis had additional changes of focal hydro-nephrosis with interstitial fibrosis. There was no case of biopsy-confirmed pre-existing renal disease in the series.

**Table 3:** Renal pathology in cases of renal tuberculosis (n=12) TB: tuberculosis

Types of renal pathology	Cases with associated lung TB (n=2)	Cases with associated abdomen TB (n=1)	Cases with no extra-renal focus of TB (n=9)	Total cases
Congestive changes in kidney	--	--	1	1
Focal tubular necrosis	1	--	--	1
Focal hydro-nephrosis with interstitial fibrosis	1	1	1	3
Glomerular sclerosis with changes in tubule (atrophy & thyroidization)	--	--	1	1
Hydropic changes in tubule	--	--	2	2
+ Presence of TB granuloma	2	1	9	12

## Discussion

The term autopsy is derived from the Greek word “autopsies” meaning auto (oneself) and opsis (eye) which is “to see for self.” Medico-legal autopsies are a mandatory specialized surgical procedure conducted on the corpse to determine the cause of death. Even in the era of high-tech medicine, the autopsy remains an important tool for the quality assessment of clinical diagnosis [1]. Various findings, unrelated to the cause of death may be noticed during histopathological examination of various organs and tissues retrieved during autopsies.

Our series of 417 cases had 64.26% males and 35.74% females. Larsen et al. in their largest retrospective series on medico-legal autopsy in Denmark had 10,252 (68.39%) males and 4738 (31.61%) females [4]. Several authors have reported male preponderance [5-7]. However, Perrone et al. in their study reported 46% males and 54% females [8]. This could be due to the fact that their study was a specially designed clinical autopsy series to find out renal diseases that go unrecognized.

The maximum number of cases (47.24%) in our series belonged to the age group of 11-30 years, followed by 36.45% cases in the age group of 31-50 years. Combined together, these groups accounted for 83.69% cases. Palemo et al. had similar age distribution with 68.4% cases of medicolegal autopsies falling in the age group of 20-49 years [5]. However, Larsen et al. had the highest number of cases in the age group of 40-59 years followed by 20-39 years age group [4]. They attributed the higher age group involvement to the demographic pattern of the population with higher elderly population.

Among the 417 autopsies, our series had 159 (38.12%) cases with definitive renal pathologies. Perrone et al. identified 58 cases (41%) with definitive histological alterations in their series of 148 cases [8]. In our series, there were predominant involvement of tubular and interstitial components in 93 (58.49%) cases and 61 (38.36%) cases where all components of renal tissue were involved. There were four (2.51%) cases with only vascular lesions and one case (0.62%) of Hodgkin’s lymphoma.

Among the 93 (58.49%) cases in our series with the predominant changes in tubular and interstitial tissue, we found hydropic degeneration in 50 cases, chronic pyelonephritis in 45 cases, tubular necrosis in 24 cases, tuberculosis in 12 cases, and chronic inflammation and abscess in seven cases. Muley et al. had tubular and interstitial lesions in a lower percentage of cases (30.90%), consisting of changes of acute tubular necrosis, chronic pyelonephritis, tubular hemorrhages, and interstitial nephritis [6]. They had renal arteriosclerosis 125 (22.7%) cases. Wen et al. after performing analysis of 292 studies (76% of studies included were single-center case reports), identified 16 histologic descriptions of tubular injury, including tubular cell sloughing (39.4%), tubular epithelial flattening/simplification (37.3%), tubular dilatation (hydropic changes) (37%) and tubular cell necrosis (31.8%) [9]. There was no difference in tubular injury histology among different tissue procurement types (native kidney biopsy, transplant kidney biopsy, and autopsy), among different etiologies, or between different tissue procurement timing. The percentage of cases with hydropic changes and those with evidence of tubular necrosis in our series is nearly similar as was found in that large analysis. This suggests that despite the relative delay in conducting autopsies in our set-ups, the changes of tubular injuries detected on histopathology are similar to those observed in the western world.

Among the 61 cases where definitive pathological changes were identifiable in all components of renal tissues, there were changes suggestive of chronic kidney diseases, glomerulosclerosis like global changes in 20 cases (32.78%), segmental changes in four (6.55%), nodular changes in six (9.83%), both global and segmental component in 27 (44.26) cases. Basement membrane thickening was present in 21 (34.42%) and glomerulonephritis with crescent formation in two (3.27%) cases. Muley et al. had higher percentage of non-glomerular nephropathies (55.04%) as compared to our series [6]. They had glomerular lesions in 14.17% cases, which exhibited glomerular alterations such as glomerular sclerosis and glomerulonephritis. Kakadiya et al. in their study found the commonest lesion being chronic pyelonephritis, followed by glomerulosclerosis and glomerulonephritis [7]. However, their study was silent about further details of pathological changes. Global changes, segmental changes, nodular changes, both global and segmental component in the same patient, crescent formation and basement membrane thickening, present in our series, has been reported by many authors. Yusuke et al. showed that the global glomerular sclerosis, interstitial fibrosis, tubular atrophy, and arteriosclerosis lesions were predominantly associated with aging and were largely independent of hypertension [10]. Despite the presence of all these features in a significant number of cases in our series, we cannot correlate our findings with the study of Yusuke et al. because 54.93% cases in our series were those who were brought dead to our institute and had no previous medical records available.

Only four (2.5%) cases in the series had isolated vascular changes (thickening of tunica media) in the renal vessels. Three of these four subjects were elderly males. Most studies confirm that atherosclerotic involvement of the intrarenal vasculature is common in the elderly population [11,12]. Kasiske in his study, on the relationship between vascular disease and age-associated changes in human kidney, demonstrated strong, independent associations between age, intrarenal vascular disease, and glomerulosclerosis [13].

Kidney involvement is an under-recognized, uncommon complication of both Hodgkin's and non-Hodgkin's lymphoma [14]. There was one case of Hodgkin’s lymphoma in our series. There are some small case series and case reports in the literature about the presence of Hodgkin's lymphoma in the renal tissue [15,16].

We had 57 cases with evidence of tuberculosis on post-mortem including 12 cases (21%) of renal tuberculosis. De Francesco Daher, in their review article, reported genitourinary tuberculosis, accounting for 27% (range, 14 to 41%) of non-pulmonary tuberculosis in the United States, Canada, and England [17]. According to them, renal involvement by tubercular infection is underdiagnosed in most health care centers. Similar observations have also been reported by many authors [18]. In our series, only two cases with renal tuberculosis had concurrent lung tuberculosis whereas one case had concurrent abdominal tuberculosis. The absence of lung lesions in renal tuberculosis in the majority of our cases is due to the fact that at the time of presentation of genitourinary TB, the lung lesions are often healed. Our observations are endorsed by many other authors [19,20].

## Conclusions

Our study demonstrated that in more than one-third (38%) cases of autopsies, there are wide spectrums of renal pathologies present which points towards possibilities of wide range of disease processes present in the body. Those renal changes may have an impact on the management of patients and may be contributing factors leading to death. The study demonstrated that the commonest changes are present in tubules and interstitial tissue followed by changes in glomeruli. Incidental findings like the presence of renal tuberculosis in a significant number of cases are worrisome as these are often missed and lead to the development of renal insufficiency, chronic renal diseases, and end-stage renal disease. Isolated vascular changes and even neoplasms, occasionally detected on histopathology are bad prognostic factors. The physician as well as forensic pathologists must be aware of the high prevalence and wide spectrum of possible pathologies in the kidney. The focus should be to develop more efficacious diagnostic methods and intervention techniques keeping into consideration the possibilities of wide spectrum of renal pathologies possible.
